# Supplementary material for: Diffusion Tensor Imaging Reveals Deep Brain Structure Changes in Early Parkinson’s Disease Patients with Various Sleep Disorders
Source: Brain Sci. 2022 Mar 30;12(4):463. doi: 10.3390/brainsci12040463 (PMC9025175; doi:10.3390/brainsci12040463)
Supplement: Supplementary file 1 [file brainsci-12-00463-s001.zip › Table S1. The mean score for the PDSS-2 and its each item in PD patients.pdf]

Table S1. The mean score for the PDSS-2 and its each item in PD patients.

	**N**	**Mean±SE**
Subitem.1	36	2.11 ± 0.206
Subitem.2	36	1.72 ± 0.202
Subitem.3	36	1.86 ± 0.170
Subitem.4	36	0.97 ± 0.141
Subitem.5	36	0.97 ± 0.116
Subitem.6	36	1.86 ± 0.211
Subitem.7	36	0.50 ± 0.141
Subitem.8	36	2.14 ± 0.160
Subitem.9	36	1.17 ± 0.180
Subitem.10	36	0.86 ± 0.150
Subitem.11	36	1.39 ± 0.188
Subitem.12	36	0.61 ± 0.145
Subitem.13	36	1.39 ± 0.208
Subitem.14	36	1.22 ± 0.183
Subitem.15	36	0.67 ± 0.149
Total of PDSS-2	36	19.44 ± 1.359
