# Supplementary figures and images for: Diffusion Tensor Imaging Reveals Deep Brain Structure Changes in Early Parkinson’s Disease Patients with Various Sleep Disorders
Source: Brain Sci. 2022 Mar 30;12(4):463. doi: 10.3390/brainsci12040463 (PMC9025175; doi:10.3390/brainsci12040463)

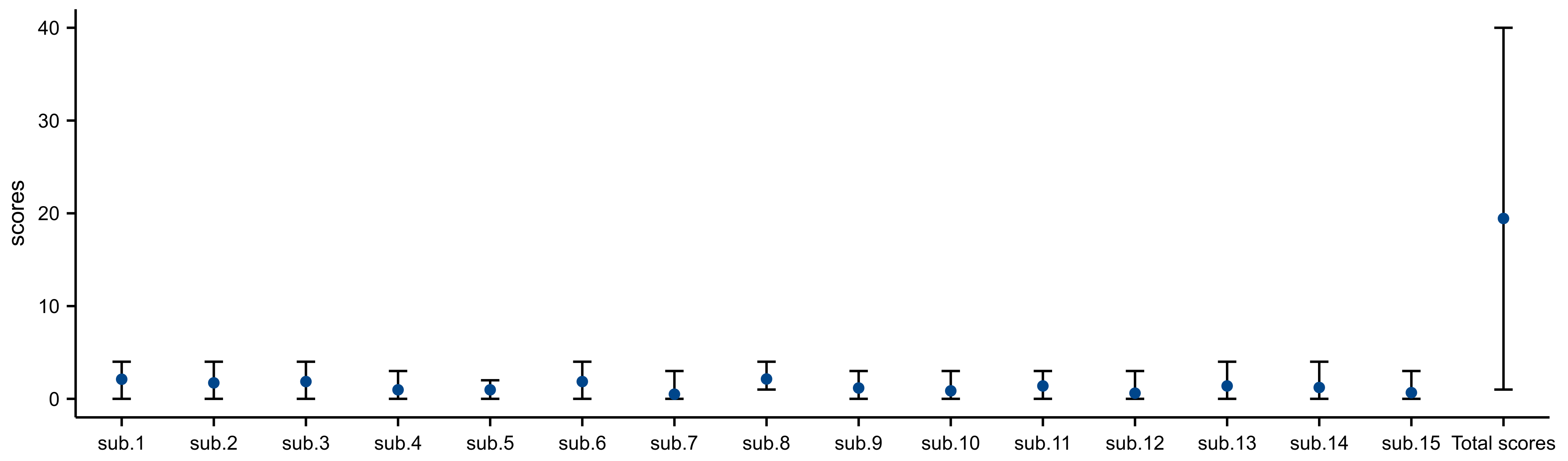

Supplement: Supplementary file 1 [file brainsci-12-00463-s001.zip › Figure S1. The mean score for the PDSS-2 and its each item in PD patients.tiff]
